# Machine Learning Models of Survival Prediction in Trauma Patients

**DOI:** 10.3390/jcm8060799

**Published:** 2019-06-05

**Authors:** Cheng-Shyuan Rau, Shao-Chun Wu, Jung-Fang Chuang, Chun-Ying Huang, Hang-Tsung Liu, Peng-Chen Chien, Ching-Hua Hsieh

**Affiliations:** 1 Department of Neurosurgery, Kaohsiung Chang Gung Memorial Hospital and Chang Gung University College of Medicine, Kaohsiung 833, Taiwan; ersh2127@cloud.cgmh.org.tw; 2 Department of Anesthesiology, Kaohsiung Chang Gung Memorial Hospital and Chang Gung University College of Medicine, Kaohsiung 833, Taiwan; shaochunwu@gmail.com; 3 Department of Trauma Surgery, Kaohsiung Chang Gung Memorial Hospital and Chang Gung University College of Medicine, Kaohsiung 833, Taiwan; jjfce0624@gmail.com (J.-F.C.); junyinhaung@yahoo.com.tw (C.-Y.H.); htl1688@yahoo.com.tw (H.-T.L.); 4 Department of Plastic Surgery, Kaohsiung Chang Gung Memorial Hospital and Chang Gung University College of Medicine, Kaohsiung 833, Taiwan; VENU_CHIEN@hotmail.com

**Keywords:** neural networks (NN), logistic regression (LR), machine learning (ML), survival, support vector machine (SVM), Trauma and Injury Severity Score (TRISS)

## Abstract

Background: We aimed to build a model using machine learning for the prediction of survival in trauma patients and compared these model predictions to those predicted by the most commonly used algorithm, the Trauma and Injury Severity Score (TRISS). Methods: Enrolled hospitalized trauma patients from 2009 to 2016 were divided into a training dataset (70% of the original data set) for generation of a plausible model under supervised classification, and a test dataset (30% of the original data set) to test the performance of the model. The training and test datasets comprised 13,208 (12,871 survival and 337 mortality) and 5603 (5473 survival and 130 mortality) patients, respectively. With the provision of additional information such as pre-existing comorbidity status or laboratory data, logistic regression (LR), support vector machine (SVM), and neural network (NN) (with the Stuttgart Neural Network Simulator (RSNNS)) were used to build models of survival prediction and compared to the predictive performance of TRISS. Predictive performance was evaluated by accuracy, sensitivity, and specificity, as well as by area under the curve (AUC) measures of receiver operating characteristic curves. Results: In the validation dataset, NN and the TRISS presented the highest score (82.0%) for balanced accuracy, followed by SVM (75.2%) and LR (71.8%) models. In the test dataset, NN had the highest balanced accuracy (75.1%), followed by the TRISS (70.2%), SVM (70.6%), and LR (68.9%) models. All four models (LR, SVM, NN, and TRISS) exhibited a high accuracy of more than 97.5% and a sensitivity of more than 98.6%. However, NN exhibited the highest specificity (51.5%), followed by the TRISS (41.5%), SVM (40.8%), and LR (38.5%) models. Conclusions: These four models (LR, SVM, NN, and TRISS) exhibited a similar high accuracy and sensitivity in predicting the survival of the trauma patients. In the test dataset, the NN model had the highest balanced accuracy and predictive specificity.

## 1. Background

Among various prediction models for mortality outcomes of trauma patients, the Trauma and Injury Severity Score (TRISS) remains the most commonly used algorithm [1,2,3]. The TRISS model was created by logistic regression (LR) in 1987 using a small cohort of a single center to predict survival probability [4]. In 1990, it was used to provide a comprehensive overview of outcomes in trauma patients by the American College of Surgeons’ Major Trauma Outcome Study (MTOS) after the evaluation of more than 80,000 patients [5]. The TRISS calculator determines the probability of survival from age as a dichotomous variable, Injury Severity Score (ISS, an anatomical variable), Revised Trauma Score (RTS, a physiological variable), and the use of different coefficients for blunt and penetrating injuries. The ISS is a measure of the severity of injury using an Abbreviated Injury Scale (AIS) [6], which scores the severity of injury on a scale of 1 to 6 for six specified anatomical regions. The sum of the squares of the three highest AIS scores is used to calculate ISS, ranging from 1 to 75 [7]. The RTS is a weighted summation of coded variable values of the patient’s initial Glasgow Coma Scale (GCS) score, systolic blood pressure (SBP) and respiratory rate (RR) [8]. 

The TRISS model has provided a good standard for survival prediction for more than 30 years and attempts to replace TRISS have failed [9]. In a review of 90 articles on prognostic models for general trauma patients, the TRISS model was externally validated in 43 articles [3]. Furthermore, there were 112 TRISS-based models that were subsequently developed [3]. However, criticism of the TRISS persisted because TRISS scores in middle ranges (25–75%) have large inconsistencies in terms of reliability [10,11]. Thus, the results of the TRISS model differ dramatically on subsets of trauma patients [12,13]. Furthermore the TRISS model, which was derived from a study based on the North American trauma population, might present unvalidated results in Europe, where a substantially lower proportion of patients suffer penetrating trauma [9], or in Asia where motorcycle or fall accidents comprise a majority of the trauma population [14,15,16].

Given that the TRISS model estimates survival probability using linear statistical models and only a small set of variables were selected for analysis, it is questionable as to whether these models are capable of analyzing complex biological systems such as traumatized humans. Furthermore, it is important to know whether the provision of additional information, including the presence of pre-existing comorbidities or laboratory data, would improve the predictive performance of TRISS [17]. Nonetheless, limitations of overfitting and multicollinearity of regression analysis may preclude the analysis of many explanatory variables. Currently, machine learning (ML) has been successfully applied to aid in clinical diagnosis and predictive prognosis [18,19,20]. Examples of ML techniques applied in a clinical setting include, but are not limited to, support vector machine (SVM), neural networks (NN), decision trees, random forest trees, and Bayes classification. SVM and NN can be used as a nonlinear statistical data modeling tool to deal with complex relationships and have shown significantly better predictive performance than LR in some illness [20,21]. In our prior study that build the ML model to predict the mortality of individual hospitalized motorcycle riders, we had found that LR and SVM outperformed the decision tree (DT) in prediction accuracy [22]. Therefore, the current study aimed to investigate whether the addition of more clinical variables and the application of the SVM or NN models can improve performance, compared to TRISS, in predicting the survival probability of trauma patients.

## 2. Methods

### 2.1. Subject and Data Preparation

This study was approved by the Institutional Review Board (IRB) of Kaohsiung Chang Gung Memorial Hospital, a 2686-bed level I trauma center in Southern Taiwan [14,15,16], with approval number 201701324B0. Because of the retrospective design study, the requirement for informed consent was waived according to the regulation of the IRB. Detailed patient information for the period January 2009 to December 2016 was retrieved from the Trauma Registry System of the hospital. Information used included age, sex, pre-existing comorbidities [coronary artery disease (CAD), congestive heart failure (CHF), cerebral vascular accident (CVA), diabetes mellitus (DM), end-stage renal disease (ESRD), hypertension (HTN), vital signs (temperature, systolic blood pressure (SBP), diastolic blood pressure (DBP), heart rate (HR), and respiratory rate (RR) which were recorded at triage upon arrival at the emergency department), Glasgow coma scale (GCS) score, AIS in different body regions, RTS, ISS, and TRISS. Blood-drawn laboratory data at the emergency room, including white blood cell count (WBC), red blood cell count (RBC), hemoglobin (Hb), hematocrit (Hct), platelets, neutrophil (%), international normalized ratio (INR), glucose, sodium (Na), potassium (K), blood urine nitrogen (BUN), creatinine (Cr), aspartate aminotransferase (AST), alanine aminotransferase (ALT) were also used. Finally, in-hospital mortality was recorded. The survival prognosis of TRISS is computed based on a logarithmic regression equation: Survival probability = 1/(1 + e-b), where b (blunt injury) = −0.4499 + 0.8085 × RTS − 0.0835 × ISS − 1.7430 × Age(index) and b (penetrating injury) = −2.5355 + 0.9934 × RTS − 0.0651 × ISS − 1.1360 × Age(index). The Age(index) value is set to 0 in the formula for patients below 55 years old, and is set to 1 for patients older than 55 [4]. Those patients who had missing laboratory data were not included in the analysis. Multiple imputation of missing values using the Markov Chain Monte Carlo simulation was applied to deal with other missing data, which accounted for less than 2% of the total. To avoid multicollinearity, variables that were closely correlated with each other (Hct and Hb, AST and ALT, SBP/DBP/RR and RTS) were excluded before being subjected to further analysis. From 2009 to 2016, all enrolled patients were divided into a training dataset (70% of the original data set) for generation of a plausible model under supervised classification, and a test dataset (30% of the original data set) to test the performance of the model. Among the enrolled 18,811 patients, 13,208 (12,871 survival and 337 mortality) and 5603 (5473 survival and 130 mortality) patients comprised the training and test datasets, respectively. An additional validation dataset was created with data randomly selected from 30% of the training dataset and 30% of the test dataset to provide an unbiased evaluation of a model fit on the training dataset. The data in the validation dataset was divided into ten parts with model hyperparameters being fine-tuned by a grid search with 100-fold cross validation to determine the values that led to the best performance and avoided over fitting.

### 2.2. Machine Learning Modeling

#### 2.2.1. Logistic Regression (LR)

The LR classifier used generalized liner model (glm) and step function in the stats package of R 3.3.3 (R Foundation for Statistical Computing, Vienna, Austria) to predict the likelihood of occurrence of survival in the trauma patients. A stepwise-selected multivariate LR was performed to identify significant predictors of survival using forward selection and backward elimination repeatedly. The Hosmer–Lemeshow test was used as a statistical test for goodness of fit for the LR models. A prediction model was established based on the sum of scores calculated from these independent risk factors and their regression coefficient.

#### 2.2.2. Support Vector Machine (SVM)

The SVM classifier used the e1071 package in R with the tune.svm and svm functions. The SVM classifier handles non-linear interactions [23] by estimating the optimal operating point using a grid search with a 10-fold cross-validation varying the penalty parameter C, which determined the tradeoff between minimization of fitting error and model complexity, and hyper-parameter γ, which defined the nonlinear feature transformation onto a higher dimensional space and controlled the tradeoff between error due to bias and variance in the model [23]. 

#### 2.2.3. Neural Networks (NN)

The NN used the Stuttgart Neural Network Simulator (RSNNS) [24] with multilayer perceptron (mlp) method and Std_Backpropagation parameter in R to create the model. The models contained three layers: an input layer, two layers of hidden nodes, and a single output node. Thirty-one input variables were applied in the current models. The number of hidden layer neurons was determined through trial and error during the selection of a predictive network with the best sensitivity and specificity. Tuning parameters included the number of nodes in the hidden layer optimized between one and 20, maximal of iterations to learn was set to 200, and other parameters were set to default for the training process. Iterations occurred until the error did not significantly decrease to avoid over-training and to enhance the capacity for generalization [25]. 

### 2.3. Predictive Performance

The accuracy, sensitivity, and specificity of the four models (LR, SVM, NN, and TRISS) were calculated. Discrimination of the models was assessed according to the area under the curve (AUC) of the receiver operating characteristic curves (ROCs) using the roc and roc.test functions in the pROC package in R, as this approach allowed for comparison of two or more empirical curves that were constructed from tests performed on the same individuals [26]. Based on a confusion matrix, the balanced accuracy is given by 1/2 × (true-positive/positive + true-negative/negative) to deal with imbalanced datasets. Somers’ Dxy rank correlation coefficient, c-index, R^2^, and Brier score of these models were calculated. Somers’ Dxy assesses the predictive discrimination with measured probability of concordance minus the probability of discordance between predicted outcomes and observed outcomes [27]. The C-index is a measure of how well the model can discriminate between those who survive and those that do not; the model is considered to have outstanding discrimination when the c-index score >0.9.

Calibration curves were used to determine the degree of agreement between predicted probabilities and observed outcomes. The R^2^ quantifies the goodness-of-fit of a model [28], with an *R*^2^ = 1 indicating that the regression line perfectly fits the data. The Brier score is defined as the mean squared difference between the predicted probability and the actual outcome and presents an overall measure of model performance [29]. Brier scores vary between 0 and 1, a lower Brier score is indicative of a better calibrated prediction.

### 2.4. Statistical Analyses

All statistical analyses were performed using SPSS 23.0 (IBM Inc., Chicago, IL, USA) or R 3.3.3. For categorical variables, we used chi-square tests to determine the significance of the association between variables. For continuous variables, we used Kolmogorov–Smirnov test to analyze the normalization of the distributed data and used Mann–Whitney *U* tests to analyze non-normally distributed data. Results are presented as median ± interquartile range (IQR). A *p*-value <0.05 was taken as statistically significant.

## 3. Results

### 3.1. Patient Demographics

Among the enrolled 18,811 patients, there were 18,344 survival and 467 mortality outcomes. Except for CVA history, there were statistically significant differences in all variables between survival and fatal patients, including age, gender, pre-existing comorbidities, HR, temperature, GCS, AIS in different body region, ISS, RTS, and blood-drawn laboratory data (Table 1 and Table 2). Thirty-one variables were used for imputation in the ML classifiers. 

### 3.2. Construction of the ML Classifiers

Logistic regression identified 19 predictors (CHF, INR, CAD, Cr, ISS, WBC, age, HR, platelets, neutrophil, Na, Hb, AIS-Thorax, GCS, HTN, temperature, AIS-Extremity, RTS, AIS-Face) as independent risk factors for survival (Appendix A Table A1), with ISS and age being the most important two features that determine the survival of an individual trauma patient (Appendix A Table A2). The SVM classifier was used to predict mortality, taking inputs from all 31 variables, with two parameters (C, γ) being determined by a grid search of 2^x^, where x is an integer between −20 and 4 for both C and γ. The values which gave the highest 100-fold cross-validation accuracy were C = 0.003906 and γ = 0.003906. The constructed NN model (RSNNS) included 31 inputs, five neurons in the first hidden layer and 18 neurons in the second hidden layer, and one output neuron. A single output node indicated the probability of survival.

### 3.3. Performance of the ML Classifiers

All four models established in the training process had an accuracy and sensitivity of more than 98% (Table 3). Because there was a higher rate of survival than mortality across the patient cohort, this fact would be accompanied by a higher accuracy and sensitivity in predicting survival. Consequently, we focused on the level of specificity of these models. NN had the highest specificity (72.7%), followed by the TRISS (71.2%), SVM (50.8%), and LR (45.1%) models. All four models had a high accuracy exceeding 97.5% and a sensitivity of more than 98.6%. For balanced accuracy in validation dataset, NN (82.0%, 95% CI: 81.8–82.2%) and the TRISS (82.0%, 95% CI: 81.6–82.0%) presented the highest score, followed by SVM (75.2%, 95% CI: 74.9–75.5%), and LR (71.8%, 95% CI: 71.5–72.1%) models. In the test dataset, NN exhibited the highest score of balanced accuracy (75.1%), followed by the TRISS (70.2%), SVM (70.6%), and LR (68.9%) models. In addition, NN exhibited the highest specificity (51.5%), followed by the TRISS (41.5%), SVM (40.8%), and LR (38.5%) models. 

**Figure 1:**
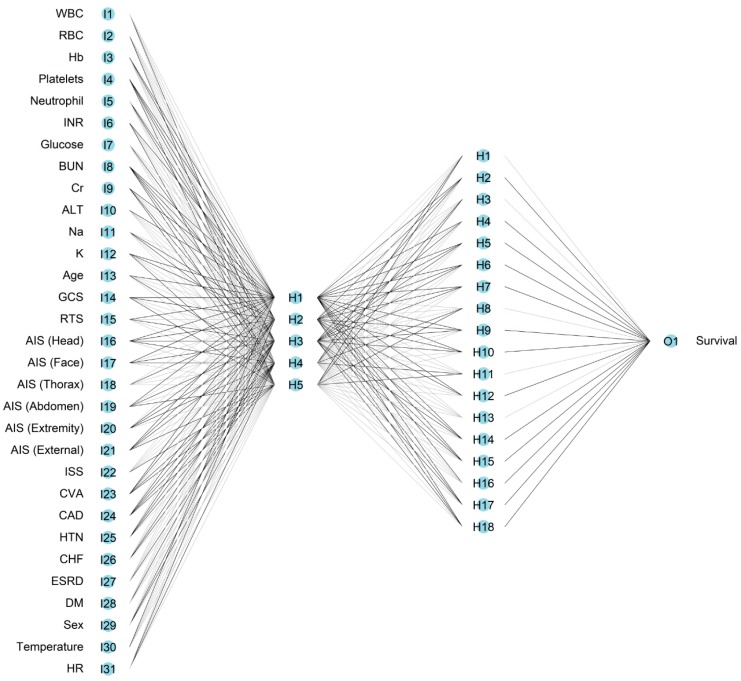
Architecture of the four-layered neural network.

A comparison of AUCs of the ROCs among these four models for the training dataset (Figure 1), demonstrated that the LR (AUC 0.967) model had a significantly higher AUC than the NN (AUC 0.959) and TRISS (AUC 0.934) models; SVM (0.964) had a significantly higher AUC than TRISS; however, there was no significant difference in AUC between LR and the SVM nor between SVM and NN. In addition, NN had a significant higher AUC than TRISS. In the test dataset, both LR (AUC 0.958) and SVM (AUC 0.964) had a significantly higher AUC than NN (AUC 0.944) and TRISS (AUC 0.930); however, there no significant difference in AUC between LR and the SVM models was apparent. In addition, NN had a significantly higher AUC than TRISS. In either the training or test datasets, the TRISS exhibited a significantly worse performance in predicting survival than the three remaining models. 

**Figure 2:**
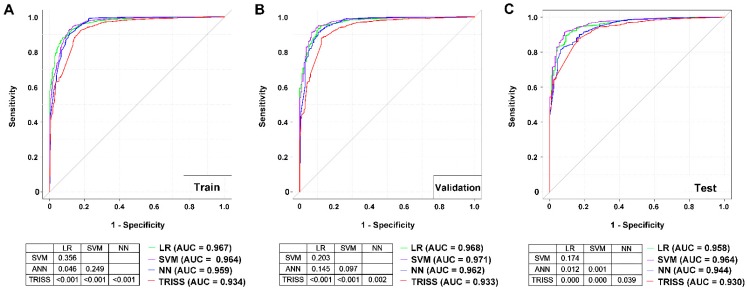
Receiver operating characteristic (ROC) curves for the LR, SVM, NN, and TRISS models in predicting the survival of trauma patients in the (**A**) train, (**B**) validation, and (**C**) test datasets.

The calibration curves of these four predictions demonstrated a non-significant miscalibration in model development, with small differences between models. The LR model generated a nonparametric line close to the ideal diagonal line with the highest *R*^2^ (0.569) and with a Brier score of 0.014 (Figure 2), while the NN had the smallest R^2^ and lowest Brier score (0.010). By contrast, the TRISS model exhibited deviation from the ideal diagonal line and there was a clear difference between the predicted probability and the actual outcome, especially for those individuals who had low predicted probability.

## 4. Discussion

More predictive variables may lead to a more accurate performance for LR models such as TRISS. In a review of 90 articles on prognostic models for the general trauma population [3], the lowest AUCs were found in a model with age and comorbidities as predictors (AUC 0.59). In the Kampala Trauma Score (KTS) (AUC 0.62), which is calculated using the patient’s age, SBP, RR, neurologic status (alert, responds to voice, responds to pain, or unresponsive), and number of serious injuries [30], the highest AUC was found for a TRISS-based model with updated coefficients based on goodness-of-fit for their own study population (AUC 0.98) [3]. Furthermore, the inclusion of dichotomized, categorical, or continuous predictors in the models may result in differences in predictive performance. The comparison of the TRISS model with TRISS-based models that incorporated different measurement levels for age or ISS [3,31,32] revealed that models with categorical variables presented better calibration and discrimination compared with dichotomized variables, and models that included continuous variables showed even better calibration and discrimination [24,31,32]. 

Theoretically, models that included predictors from all categories (physiological, anatomical, and demographic variables, and injury cause/mechanism) show better discrimination compared to models incorporating only one or two categories. However, systematic reviews have demonstrated that adding more predictors to the basic TRISS-model did not always result in better performance [33,34]. Although more predictive variables may lead to a more accurate performance, the complex interaction among these independent and dependent variables may hinder their predictive performance. It is also recognized that with the increased number of potential risk factors, the complexity of the models can cause over-fitting and yield implausible results. The TRISS-based models included acute ethanol poisoning as an additional predictor showed even worse discriminatory power [35]. Furthermore, the variable chosen as an input to the model should be practical. For example, base deficit could be an important predictor for mortality for trauma patients. However, base deficit is mostly assessed only in severely injured patients [36] and often would not be recorded and would therefore be presented as a missing value in the general trauma population. Therefore, it is not practical to incorporate base deficit into the model to predict mortality for the general trauma population. Likewise, as more input variables are included in the model, the problem of increasing rates of missing values becomes difficult to overcome [3]. 

One study used 16 anatomical and physiological predictor variables to predict mortality outcomes in 10,609 trauma patients and revealed that the performance of the NN (AUC 0.912) exceeded that of the TRISS (AUC 0.895) model [37]. The NN model was more accurate and outperformed the LR model in predicting in-hospital mortality for patients in critical care [38] and under mechanical ventilation [39]. In this study, although SVM and NN were both expected to handle complex nonlinear relationships between independent and dependent variables better than LR [40,41], the SVM outperformed the NN in the test dataset, and there was no significant difference in AUC values between the LR and SVM. The performance of an ANN depends on the number of parameters, network weights, the selection of an appropriate training algorithm, the type of transfer functions used, and the determination of network size [42]. Neural networks require initialization and adjustment of many individual parameters to optimize the performance of the classification. The NN model was developed empirically and can be over-fitted for training data [42]. Many researchers have compared NN versus LR models and found that NN and LR models have similar classification performance [42]. Nonetheless, inferences about the explanatory variables of NN are more difficult to interpret than those derived from LR analysis [43]. In addition, the employment of kernels in SVM help the model learn non-linear decision boundaries, allowing the classifier to solve more complex data than linear analytic methods [44] and the SVM boundary is only minimally influenced by outliers [45].

Some limitations of this study include the following: First, patients declared dead on arrival at the emergency room were not recorded in the registered database and this may have resulted in a selection bias [15,16]. Furthermore, because the registered trauma data included only in-hospital mortality but no information regarding mortalities at 30 days or a longer, additional selection biases in assessing mortality may be present. Second, we included more variables, such as preexisting comorbidities and laboratory data, to improve prediction performance. However, collection of physiological and laboratory data at the time of arrival at the emergency department may not reflect further changes in hemodynamics and metabolic variables of patients who were under possible management or resuscitation. The lack of specific mortality-related information, including resuscitation or mechanism of trauma may have limited the accuracy of the prediction model. Third, this study was unable to assess the effects of any one particular treatment intervention, especially surgical interventions to the patients. We can only assume that these treatments were uniform across the population data. Fourth, our analysis used only single-center data obtained in southern Taiwan, which may not be representative of other populations. Further, in the study, we had chosen the RSNNS as one representative of NN models because it performed better than other ANN packages in R (data not shown). Although the results of the NN model seemed unimpressive, we believe that NN models may still be valuable in predicting the outcomes of trauma because NN algorithms are evolving quickly. Further, although the addition of more clinical variables or the application of the SVM or NN models can significantly improve the predictive performance compared to that of TRISS, the improvement was modest. It may be argued that it may not be worth getting such improvements by using much more complex input variables than the TRISS model. However, we believe that in the future, real-time processing of monitored patient data will reduce the burden of such laborious works. In addition, random forests is an important class of ML models with a different conception than the other models. Whether it can improve the predictive performance is worthy for further investigation but not explored in this study. Finally, because of low specificity of above ML models in predicting survival, an ensemble of regressors from these different models may be worthy to be built to test whether it can improve the predictive power.

## 5. Conclusions

In this study, we demonstrated that these four models (LR, SVM, NN, and TRISS) exhibited a similar high accuracy and sensitivity in predicting the survival of the trauma patients. In addition, in the test dataset, the NN model had the highest balanced accuracy and predictive specificity out of the four models tested. The results of this study may provide encouraging information for the development of a new NN prediction model that can be integrated into trauma care systems to predict survival of trauma patients.

## Figures and Tables

**Table 1:** Continuous variables of patient characteristics of those trauma patients who survived or not.

Variables	Total	Survival		*P*-Value
	(*n =* 18,811)	No (*n =* 467)	Yes (*n =* 18,344)	
Age (years)	51 (30, 66)	63 (44, 76)	50 (30, 66)	<0.001
HR (times/min)	86 (75, 98)	95 (78, 114)	86 (75, 98)	<0.001
Temperature (°C)	36.5 (36.2, 36.9)	36.2 (36.0, 36.7)	36.5 (36.2, 36.9)	<0.001
WBC (10^3^/uL)	10.6 (8.1, 14.1)	12.8 (9.2, 18.1)	10.6 (8.1, 14.0)	<0.001
RBC (10^6^/uL)	4.5 (4.1, 4.9)	4.0 (3.4, 4.6)	4.5 (4.1, 4.9)	<0.001
Hb (g/dL)	13.3 (12.0, 14.6)	11.9 (10.2, 13.9)	13.3 (12.0, 14.6)	<0.001
Platelets (10^3^/uL)	216.0 (177.0, 260.0)	180.0 (135.0, 227.0)	217.0 (178.0, 260.0)	<0.001
Neutrophil (%)	77.7 (66.9, 85.0)	76.7 (60.8, 85.0)	77.70 (67.00, 85.00)	0.014
INR	1.0 (1.0, 1.1)	1.1 (1.0, 1.3)	1.0 (1.0, 1.1)	<0.001
Glucose (mg/dL)	128.0 (111.0, 158.0)	186.0 (141.5, 253.5)	128.0 (111.0, 156.0)	<0.001
Na (mEq/L)	139.0 (137.0, 140.0)	138.0 (136.0, 141.0)	139.0 (137.0, 140.0)	0.007
K (mEq/L)	3.7 (3.5, 4.0)	3.6 (3.1, 4.0)	3.7 (3.5, 4.0)	<0.001
BUN (mg/dL)	13.0 (10.0, 17.0)	16.0 (12.0, 22.0)	13.0 (10.0, 17.0)	<0.001
Cr (mg/dL)	0.8 (0.7, 1.0)	1.0 (0.8, 1.4)	0.8 (0.7, 1.0)	<0.001
ALT (U/L)	23.0 (17.0, 37.0)	27.0 (17.0, 48.5)	23.0 (17.0, 36.0)	<0.001
GCS	15 (15, 15)	6 (3, 13)	15 (15, 15)	<0.001
ISS	9 (4, 10)	25 (16, 29)	9 (4, 10)	<0.001
RTS	8 (8, 8)	5.9 (4.1, 6.9)	7.8 (7.8, 7.8)	<0.001
TRISS	1.0 (1.0, 1.0)	0.68 (0.36, 0.91)	0.98 (0.97, 1.00)	<0.001

ALT: alanine aminotransferase; AST: Aspartate transaminase; BUN: blood urea nitrogen; Cr: creatinine; GCS: Glasgow coma scale; Hb: hemoglobin; Hct: hematocrit; INR: international normalized ratio; K: potassium; Na: sodium; ISS: injury severity score; RBC: red blood cells; WBC: white blood cells. These continuous data are expressed with median and interquartile range.

**Table 2:** Categorical variables of patient characteristics of those trauma patients who survived or not.

Variables		Total	Survival		*P*-Value
		(*n =* 18,811)	No (*n =* 467)	Yes (*n =* 18,344)	
Sex	Female	7817 (41.6%)	168 (36.0%)	7649 (41.7%)	0.015
	Male	10,994 (58.4%)	299 (64.0%)	10,695 (58.3%)	
CVA	No	18,116 (96.3%)	443 (94.9%)	17,673 (96.3%)	0.121
	Yes	695 (3.7%)	24 (5.1%)	671 (3.7%)	
HTN	No	14,011 (74.5%)	308 (66.0%)	13,703 (74.7%)	<0.001
	Yes	4800 (25.5%)	159 (34.1%)	4641 (25.3%)	
CAD	No	18,203 (96.8%)	430 (92.1%)	17,773 (96.9%)	<0.001
	Yes	608 (3.2%)	37 (7.9%)	571 (3.1%)	
CHF	No	18,664 (99.2%)	459 (98.3%)	18,205 (99.2%)	0.04
	Yes	147 (0.8%)	8 (1.7%)	139 (0.8%)	
ESRD	No	18,493 (98.3%)	436 (93.4%)	18,057 (98.4%)	<0.001
	Yes	318 (1.7%)	31 (6.6%)	287 (1.6%)	
DM	No	16,278 (86.5%)	380 (81.4%)	15,898 (86.7%)	0.001
	Yes	2533 (13.5%)	87 (18.6%)	2446 (13.3%)	
AIS (Head)	0	13,511 (71.8%)	83 (17.8%)	13,428 (73.2%)	<0.001
	1	1119 (6.0%)	12 (2.6%)	1107 (6.0%)	
	2	463 (2.5%)	9 (1.9%)	454 (2.5%)	
	3	1490 (7.9%)	31 (6.6%)	1459 (8.0%)	
	4	1711 (9.1%)	102 (21.8%)	1609 (8.8%)	
	5	502 (2.7%)	217 (46.5%)	285 (1.6%)	
	6	15 (0.1%)	13 (2.8%)	2 (0.0%)	
AIS (Face)	0	15,921 (84.6%)	402 (86.1%)	15,519 (84.6%)	<0.001
	1	961 (5.1%)	13 (2.8%)	948 (5.2%)	
	2	1885 (10.0%)	47 (10.1%)	1838 (10.0%)	
	3	44 (0.2%)	5 (1.1%)	39 (0.2%)	
AIS (Thorax)	0	16,587 (88.2%)	359 (76.9%)	16,228 (88.5%)	<0.001
	1	383 (2.0%)	11 (2.4%)	372 (2.0%)	
	2	538 (2.9%)	10 (2.1%)	528 (2.9%)	
	3	904 (4.8%)	45 (9.6%)	859 (4.7%)	
	4	376 (2.0%)	35 (7.5%)	341 (1.9%)	
	5	22 (0.1%)	6 (1.3%)	16 (0.1%)	
	6	1 (0.0%)	1 (0.2%)	0 (0.0%)	
AIS (Abdomen)	0	17,531 (93.2%)	409 (87.6%)	17,122 (93.3%)	<0.001
	1	109 (0.6%)	2 (0.4%)	107 (0.6%)	
	2	623 (3.3%)	25 (5.4%)	598 (3.3%)	
	3	377 (2.0%)	14 (3.0%)	363 (2.0%)	
	4	135 (0.7%)	15 (3.2%)	120 (0.7%)	
	5	36 (0.2%)	2 (0.4%)	34 (0.2%)	
AIS (Extremity)	0	5259 (28.0%)	307 (65.7%)	4952 (27.0%)	<0.001
	1	1243 (6.6%)	9 (1.9%)	1234 (6.7%)	
	2	7080 (37.6%)	76 (16.3%)	7004 (38.2%)	
	3	5186 (27.6%)	63 (13.5%)	5123 (27.9%)	
	4	36 (0.2%)	9 (1.9%)	27 (0.2%)	
	5	7 (0.0%)	3 (0.6%)	4 (0.0%)	
AIS (External)	0	16,627 (88.4%)	417 (89.3%)	16,210 (88.4%)	<0.001
	1	1835 (9.8%)	28 (6.0%)	1807 (9.9%)	
	2	187 (1.0%)	2 (0.4%)	185 (1.0%)	
	3	88 (0.5%)	0 (0.0%)	88 (0.5%)	
	4	17 (0.1%)	1 (0.2%)	16 (0.1%)	
	5	37 (0.2%)	9 (1.9%)	28 (0.2%)	
	6	20 (0.1%)	10 (2.1%)	10 (0.1%)	

AIS: abbreviated injury scale; CAD: coronary artery disease; CHF: congestive heart failure; CVA: cerebral vascular accident; DM: diabetes mellitus; ESRD: end-stage renal disease; HTN: hypertension.

**Table 3:** Survival prediction performance (i.e., accuracy, sensitivity, and specificity) for the logistic regression (LR), support vector machine (SVM), neural network (NN), and The Trauma and Injury Severity Score (TRISS) models in the train, validation, and test datasets.

Models		Train	Validation	Test
LR	Accuracy (95% CI)	98.2%	97.9% (97.7–98.1%)	97.8%
	Balanced Accuracy (95% CI)	72.3%	71.8% (71.5–72.1%)	68.9%
	Sensitivity (95% CI)	99.6%	99.5% (99.4–99.6%)	99.3%
	Specificity (95% CI)	45.1%	44.1% (41.1–47.1%)	38.5%
SVM	Accuracy (95% CI)	98.2%	98.0% (97.8–98.2%)	97.8%
	Balanced Accuracy (95% CI)	75.1%	75.2% (74.9–75.5%)	70.6%
	Sensitivity (95% CI)	99.5%	99.4% (99.3–99.5%)	99.2%
	Specificity (95% CI)	50.8%	51.0% (47.9–54.1%)	40.8%
NN	Accuracy (95% CI)	98.7%	98.3% (98.1–98.5%)	97.5%
	Balanced Accuracy (95% CI)	86.1%	82.0% (81.8–82.2%)	75.1%
	Sensitivity (95% CI)	99.4%	99.3% (99.4–99.5%)	98.6%
	Specificity (95% CI)	72.7%	64.4% (62.7–66.1%)	51.5%
TRISS	Accuracy (95% CI)	99.0%	98.5% (98.3–98.7%)	97.6%
	Balanced Accuracy (95% CI)	85.5%	82.0% (81.6–82.0%)	70.2%
	Sensitivity (95% CI)	99.8%	99.5% (99.4–99.6%)	98.9%
	Specificity (95% CI)	71.2%	64.5% (62.8–66.2%)	41.5%

## Appendix A

**Table A1:** The independent risk predictors factors and the coefficient in LR model.

Variables	Coefficient
Intercept	−19.2709
CHF	−1.040
INR	−0.747
CAD	−0.549
Cr	−0.182
ISS	−0.095
WBC	−0.069
Age	−0.040
HR	−0.012
Platelets	0.005
neutrophil	0.027
Na	0.065
Hb	0.124
AIS-Thorax	0.127
GCS	0.183
HTN	0.253
Temperature	0.293
AIS-Extremity	0.324
RTS	0.384
AIS-Face	0.424

**Table A2:** The importance of predictive features in LR model.

	Odds Ratio	2.5%	97.5%	Importance
ISS	0.91	0.90	0.92	12.40
Age	0.96	0.95	0.97	8.68
AIS-Extremity	1.38	1.23	1.56	5.28
Cr	0.83	0.78	0.90	5.05
neutrophil	1.03	1.02	1.04	4.85
WBC	0.93	0.91	0.96	4.82
Platelets	1.01	1.00	1.01	4.47
INR	0.47	0.34	0.67	4.41
GCS	1.20	1.10	1.31	3.96
AIS-Face	1.53	1.24	1.90	3.92
Na	1.07	1.03	1.10	3.78
HR	0.99	0.98	0.99	3.65
Hb	1.13	1.06	1.21	3.64
Temperature	1.34	1.10	1.64	2.85
RTS	1.47	1.11	1.94	2.71
AIS-Thorax	1.14	1.01	1.28	2.11
CHF	0.35	0.14	1.15	1.93
CAD	0.58	0.34	1.03	1.93
HTN	1.29	0.91	1.83	1.42
